# Diagnostic value of real-time four-dimensional transesophageal echocardiography on the implant-related thrombus

**DOI:** 10.3389/fcvm.2023.1018877

**Published:** 2023-01-26

**Authors:** Yi Yu, Rui Zhang, Yu-Han Chen, Ting Wang, Xiao-Li Tang, Chang-qi Gong, Yun Shao, Zheng Wang, Yue-Peng Wang, Yi-Gang Li

**Affiliations:** ^1^Department of Cardiology, Xinhua Hospital Affiliated to School of Medicine, Shanghai Jiao Tong University, Shanghai, China; ^2^Department of Rehabilitation Medicine, Shanghai Sixth People’s Hospital, Shanghai, China

**Keywords:** implant-related thrombus, real-time four-dimensional transesophageal echocardiography, left atrial appendage closure, cardiac implantable electronic device, imaging

## Abstract

**Objectives:**

This study aims to evaluate the diagnostic value of real-time four-dimensional transesophageal echocardiography (RT4D-TEE) for implant-related thrombus (IRT).

**Methods:**

We collected 1,125 patients with atrial fibrillation from May 2019 to February 2022 in our hospital. All patients accepted transesophageal echocardiography (TEE) examination to exclude any thrombi before the LAAC procedure.

**Results:**

There were 760 patients with LAAC, 66 patients with CIED, and 299 patients without any implantations. A total of 40 patients with an established diagnosis of IRT were further analyzed. The accurate detection rate of IRT by RT4D-TEE was 4.8% (40/826), which was higher than 3.8% (31/826) by 2D-TEE (*P* = 0.004). No IRT was found on TEE in the rest of the 786 patients. These 40 patients were divided into LAAC (*n* = 23) and CIED (*n* = 17) groups according to the results of RT4D-TEE. In the LAAC group, IRT distributed on different parts of the LAA occluder surface, 91.3% (21/23) with clumps of thrombi, and 8.7% (2/23) with a thin layer of thrombi covering the surface of the occluder. In the CIED group, thrombi were seen attached to the leads in the right atrium and right ventricle. The thrombi were beaded in 17.6% (3/17), corded in 17.6% (3/17), and clotted in the remaining 64.7% (11/17) of cases. After adjusting the anticoagulant dosage and following up for 6 months, 20% (8/40) of cases were successfully resolved, 67.5% (27/40) became smaller, and 12.5% (5/40) showed no changes.

**Conclusion:**

The accurate detection rate of IRT by RT4D-TEE was significantly higher than that by 2D-TEE. 2D-TEE has limitations, but RT4D-TEE can be used as an effective complementary method. Imaging and some clinical features differ significantly between IRT on occluder and IRT on CIED lead.

## 1. Introduction

Transcatheter left atrial appendage closure (LAAC) devices and cardiac implantable electronic device (CIED) are both increasingly used in clinical practice. A potentially fatal complication following the device implantation is thrombus formation (1–4). The majority of patients with implant-related thrombus (IRT) are diagnosed by some imaging methods during early routine follow-up (1). Timely intervention by increasing the dosage of anticoagulants could usually result in excellent outcomes. Two-dimensional transesophageal echocardiography (2D-TEE) and computed tomography have been adopted for the diagnosis of IRT (4–8). Two-dimensional transesophageal echocardiography has been reported to be a common and useful tool for diagnosis (9–11). However, the incidence of IRT is underestimated by 2D-TEE due to some missed diagnoses (4, 5, 12, 13). Real-time four-dimensional transesophageal echocardiography (RT4D-TEE) is an appealing alternative because of its superior characteristics, such as high spatial resolution, multiplanar capabilities, four-dimensional viewing, and independent of the discrepancy among different operators. Nevertheless, there has been no study using RT4D-TEE for the diagnosis of IRT on occluder and CIED lead. Moreover, differences in their IRT features between 2D-TEE and 4D-TEE have not been systematically demonstrated. Our study aims to assess the value of RT4D-TEE in detecting IRT and to summarize the echocardiographic features of IRT.

## 2. Materials and methods

### 2.1. Patients

This was a retrospective single-center study of consecutive patients who underwent LAAC with either the Watchman nitinol cage device (Atritech, Boston Scientific, Natick, Massachusetts) or LACbes device (Pushi, Shanghai), and/or CIED, including pacemaker, implantable cardioverter defibrillator (ICD), and cardiac resynchronization therapy defibrillator (CRT-D) at Xinhua Hospital from May 2019 to February 2022. Recruited patients eligible for LAAC and CIED were selected according to appropriate local and European guidelines (7). The study was approved by the Ethics Committee of Xinhua Hospital affiliated with Shanghai Jiao Tong University School of Medicine (No. XHEC-D-2022-073) and complies with the Declaration of Helsinki.

The echocardiographic data were collected from records of follow-up during the same period. All patients underwent transthoracic echocardiography (TTE) and TEE examinations. Patients who received LAAC were scheduled for routine follow-ups with TEE after the procedures. TEE examinations were also performed for patients with CIED who had new-onset AF or recurrent AF to exclude the thrombi prior to the establishment of further therapy or to assess the suspected thrombus attached to the leads by TTE in detail as indicated. TTE and TEE were performed on a commercially available system (SC2000, Siemens Healthcare, Forchheim, Germany) equipped with broadband 4V1 and Z6Ms transducers. Imaging of 2D-TEE and 4D-TEE was collected from each patient during the course of the examination, especially for the LAAC device and/or CIED lead in the 4D mode in one to three cardiac cycles. Echocardiographic experts analyzed the 2D-TEE images first and then the 4D-TEE images to confirm the diagnosis. In case of disagreement in diagnosis occurred between the two echocardiographers, a third experienced cardiologist was invited to make diagnoses until reaching a consensus. IRT by TEE was defined as a homogenous echo-dense mass visible in multiple planes with independent motion and adherence to the atrial surface of the LAAC device (1, 14). Thrombus formation in patients with CIED was defined as the aforementioned description and adherence to the CIED lead (5). The therapeutic decision to initiate anticoagulation or adjust preexisting anticoagulation was predominantly based on findings from TEE.

Antithrombotic therapy following LAAC consisted of oral anticoagulation (OAC), single antiplatelet therapy (APT), dual antiplatelet therapy (DAPT), or OAC plus APT for 1–6 months. The team of physicians performing follow-up visits gave a general recommendation to patients after CIED. Other indications were necessitated (i.e., percutaneous coronary intervention, PCI). Decisions on the duration of therapy were made according to the physician’s judgment (15).

### 2.2. Statistical analysis

Statistical analysis was performed using SPSS 26.0 (v16.0, IBM Corp., Armonk, NY) software. Continuous data were described as mean ± standard deviation (SD) and as counts and percentages if categorical. Differences in continuous and categorical variables were assessed by the chi-square test or Fisher’s exact test (if the expected value in any cell was <5), respectively. Normally distributed continuous variables were assessed by independent samples *t*-test. A *P*-value of <0.05 was considered statistically significant.

## 3. Results

**FIGURE 1:**
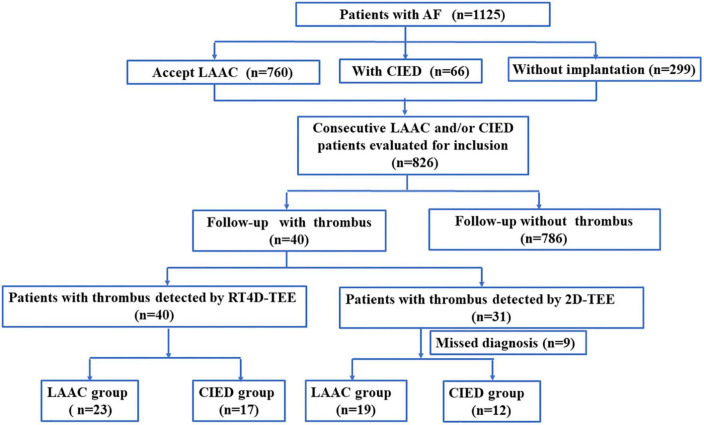
Flowchart of the study group.

We collected 1,125 patients with AF from May 2019 to February 2022 in our hospital. All patients accepted the TEE examination to exclude any thrombi. There were 760 patients with LAAC, 66 patients with CIED, and 299 patients without any implantations. A total of 40 patients with an established diagnosis of IRT were further analyzed. The accurate detection rate of IRT by RT4D-TEE was 4.8% (40/826), which was higher than 3.8% (31/826) by 2D-TEE (*P* = 0.004). No IRT was found on TEE in the rest of the 786 patients. These 40 patients were divided into LAAC (*n* = 23) and CIED (*n* = 17) groups according to the results of RT4D-TEE (Figure 1).

(1) The characteristics of the patients with IRT

The general characteristics of the 40 patients identified with IRT are summarized in Table 1. There were no significant differences in age, gender distribution, or any other recorded clinical variable. The right atrium (RA) was dilated with different degrees of tricuspid regurgitation (TR) by echocardiography. Values of these parameters except the volume of RA were significantly higher in the CIED group compared with the LAAC group (*P* < 0.05). The time history of device implantation was in different patients. It was 97.12 ± 82.14 months from implantation to the end of data collection in the CIED group and 29.20 ± 20.46 months during the same period in the LAAC group.

**TABLE 1:** Clinical characteristics of the patients with implant-related thrombus.

Variables	LAAC group (*n* = 23)	CIED group (*n* = 17)	t/x^2^	*P*-value
Age, $\bar{x}$ ± s, y	70.96 ± 7.18	69.82 ± 10.60	-0.381	0.706
Sex, *n* (%)			1.153	0.284
Male, *n* (%)	16/23 (69.57)	9/17 (52.94)		
Female, *n* (%)	7/23 (30.43)	8/17 (47.06)		
**Comorbidities and medical history**				
History of LAA thrombus, *n* (%)	2/23 (8.70)	2/17 (11.77)		
Prior PCI, *n* (%)	1/23 (4.38)	3/17 (17.65)		
Prior stroke, *n* (%)	1/23 (4.38)	2/17 (11.77)		
systemic embolism, *n* (%)	2/23 (8.70)	0		
**2D-TTE**				
LAV, ml	66.83 ± 21.29	75.61 ± 45.96	0.809	0.423
RAV, ml	42.94 ± 12.36	65.82 ± 30.36	3.277	0.002
LVEF, $\bar{x}$ ± *s*,%	59.78 ± 6.29	57.12 ± 10.03	-0.961	0.346
CHA_2_DS_2_-VASc Score, $\bar{x}$ ± s	3.35 ± 1.19	3.47 ± 1.97	0.228	0.822
HAS-BLED score, $\bar{x}$ ± s	3.17 ± 1.11	3.82 ± 2.19	1.122	0.274
Anticoagulant therapy				0.001
No OAC, no APT, *n* (%)	–	8/17 (47.06)		0.001
Single APT, *n* (%)	1/23 (4.38)	–		1.000
Dual APT, *n* (%)	–	2/17 (11.77)		0.340
OAC, no APT, *n* (%)	21/23 (91.30)	6/17 (35.29)		0.001
OAC plus APT, *n* (%)	1/23 (4.38)	1/17 (5.88)		1.000
INR, $\bar{x}$ ± s	1.12 ± 0.26	1.05 ± 0.13	-1.029	0.311

LAAC, left atrial appendage closure; CIEDs, cardiac implantable electronic devices; MI, myocardial infarction; PCI, percutaneous coronary intervention; 2D-TTE, two-dimensional transthoracic echocardiography; LAV, left atrial volume; RAV, right atrium volume; LVEF, left ventricular ejection fraction; OAC, oral anticoagulation; APT, antiplatelet therapy; INR, international normalized ratio.

(2) The characteristics of IRT in patients detected by RT4D-TEE, compared with that of 2D-TEE

In total 40 patients were precisely diagnosed with IRT by RT4D-TEE, while 77.5% (31/40) of patients were directly diagnosed with 2D-TEE. The characteristics of IRT in patients detected by RT4D-TEE are revealed in Table 2. A total of 18 out of 23 (78.26%) patients with IRT were diagnosed by 2D-TEE in the LAAC group and 5 out of 23 (21.74%) patients missed diagnosis. In addition, 13 out of 17 (76.47%) patients with IRT were diagnosed by 2D-TEE in the CIED group and 4 out of 17 (23.53%) patients missed diagnosis. Two patients with thrombosis attached to the leads were confirmed by RT4D-TEE, which were suspected of thrombus by TTE. There were no significant differences in the rate of IRT missed diagnosing by 2D-TEE between the two groups (*P* = 0.893).

**TABLE 2:** Thrombus diagnosed by real time four-dimensional Transoesophageal echocardiography.

Variables	LAAC group (*n* = 23)	CIED group (*n* = 17)	*P*- value
Optimal implantation placement, *n* (%)			0.248
Yes	20/23 (86.96)	17/17 (100.00)	
No	3/23 (13.04)		
LAA device-related thrombus, *n* (%)	23/23 (100.00)	–	
**Position of thrombus**			
Center, *n* (%)	3/23 (13.04)	–	
Periphery, *n* (%)	16/23 (69.57)	–	
Edge, *n* (%)	2/23 (8.70)	–	
Thin layer covering the surface, *n* (%)	2/23 (8.70)	–	
**Peri-device leakage**			
<5 mm, *n* (%)	7/23 (30.44)	–	
>5 mm, *n* (%)	1/23 (4.35)	–	
Residual leakage through the fabric, *n* (%)	3/23 (13.04)	–	
Incomplete endothelization, *n* (%)	10/23 (43.48)	–	
CIEDs-related thrombosis, *n* (%)	–	17/17 (100.00)	
**Position of thrombus**			
RA and RV lead, *n* (%)	–	5/17 (29.41)	
RA and RAA leads, *n* (%)	–	2/17 (11.77)	
RA lead, *n* (%)	–	7/17 (41.18)	
RV lead, *n* (%)	–	1/17 (5.88)	
RA lead and in LCS, *n* (%)	–	1/17 (5.88)	
RA lead and in LAA, *n* (%)	–	1/17 (5.88)	
Atrial septal puncture closed, *n* (%)	19/23 (82.61)	–	
6-month follow-up			0.002
Thrombus resolved, *n* (%)	7/23 (30.44)	1/17 (5.88)	
Thrombus become small, *n* (%)	16/23 (69.57)	11/17 (64.71)	
Thrombus without change, *n* (%)	0	5/17 (29.41)	

LAA, left atrial appendage; RA, right atrium; RV, right ventricular; RAA, right atrial appendage; LCS:, left coronary sinus.

**FIGURE 2:**
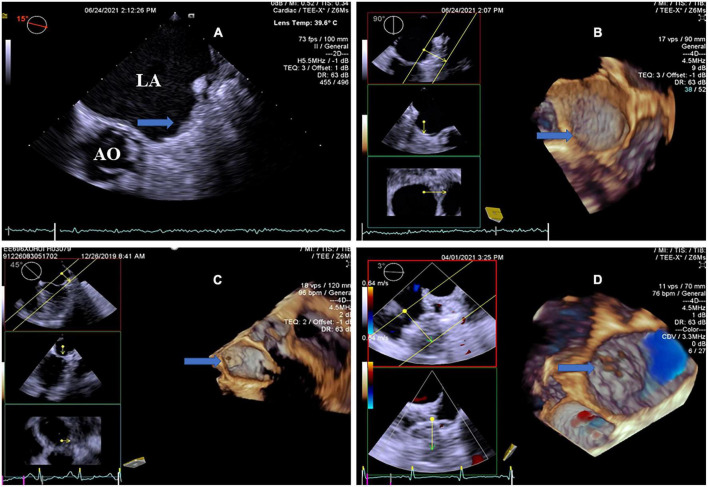
Transesophageal echocardiography follow-up images after LAAC showing optimal device placement and device-related thrombus. **(A)** 2D-TEE showed an echo-dense mass attached to the LACbes device, occupying most area of the occluder (arrow). Note severe spontaneous echo contrast throughout the left atrium. **(B)** RT4D-TEE demonstrated a thin layer of thrombi covering the whole surface of the well-seated LACbes device (arrow) after LAAC, as compared with 2D-TEE. **(C)** RT4D-TEE indicated complete LAA closure with the Watchman device, meanwhile demonstrating a mobile thrombus pedunculated with a neck attached to the occluder (arrow). **(D)** RT4D-TEE detected optimal device placement and a couple of scattered clots on the surface of the Watchman occluder (arrow). There was no residual peri-device leak at the LAA ostium detected by color Doppler. RT4D-TEE: Real-time four-dimensional echocardiography. LAAC, left atrial appendage closure; 2D-TEE, two-dimensional transesophageal echocardiography.

In the LAAC group, we found that most of the IRT originated from the periphery. IRT was evaluated with RT4D-TEE in 13.0% (3/23) of cases at the top of the center, 69.6% (16/23) at the periphery, 8.7% (2/23) at the edge, and 8.7% (2/23) with a thin layer of thrombi covering the surface of LAA occluder (Figure 2 and Supplementary Video 1). IRT was described as laminar in 8.7% (2/23), pedunculated in 8.7% (2/23), and massed in 82.6% (19/23) of cases. Compared with 2D-TEE, four patients missed the diagnosis. RT4D-TEE found more scattered IRT on the surface of the occluder in three patients and showed more clearly than that of 2D-TEE. One patient with moderate mitral regurgitation was detected by 2D-TEE, the beam rushed to the surface of the occluder, which masked the thrombus. Here, only three patients had failed to cover the rim of the left atrial appendage (LAA) with significant thrombi on the edge of the device because the occlusive device selected was too small. The mobile thrombus was seen only in two patients who pedunculated and had a neck attached to the fabric (Supplementary Video 2), and a sessile thrombus was seen in the other 19 patients. Residual leakage into the LAA was observed in five patients at the time of thrombus detection. A major peri-device leak (>5 mm) was detected in 4.4% (1/23) of patients during TEE follow-up, and a small peri-device leak (flow <5 mm) was revealed in 30.4% (7/23) of patients. Residual leakage into the LAA through the fabric was found in 13.0% (3/23) of patients. Ten patients with incomplete endothelialization have been observed.

**FIGURE 3:**
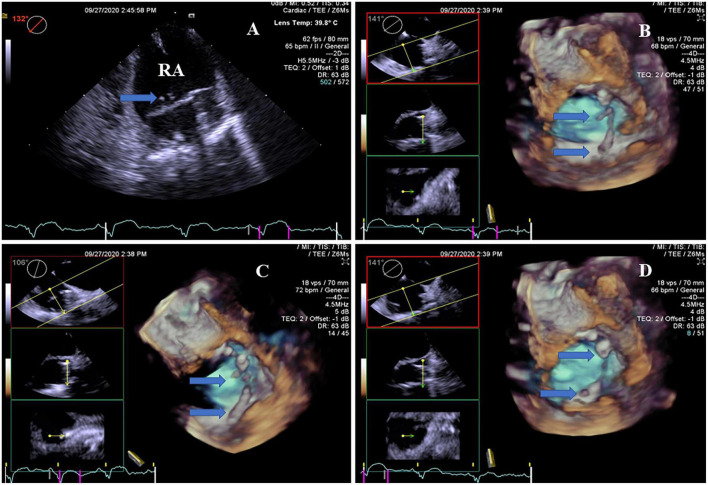
Real-time four-dimensional-TEE evaluating the pacemaker lead-related thrombus, as compared with 2D-TEE. **(A)** 2D-TEE showed two clots stuck to the lead of the pacemaker in the right atrium (arrow). Note severe spontaneous echo contrast throughout the right atrium. **(B)** RT4D-TEE demonstrated more clots attached to the lead of the pacemaker in the right atrium and more clearly than 2D-TEE (arrow). **(C)** RT4D-TEE showed more scatter clots on the lead of the pacemaker in the right atrium from a different angle (arrow). **(D)** RT4D-TEE revealed more lead-related thrombi throughout the lead of the pacemaker in the right atrium.

**FIGURE 4:**
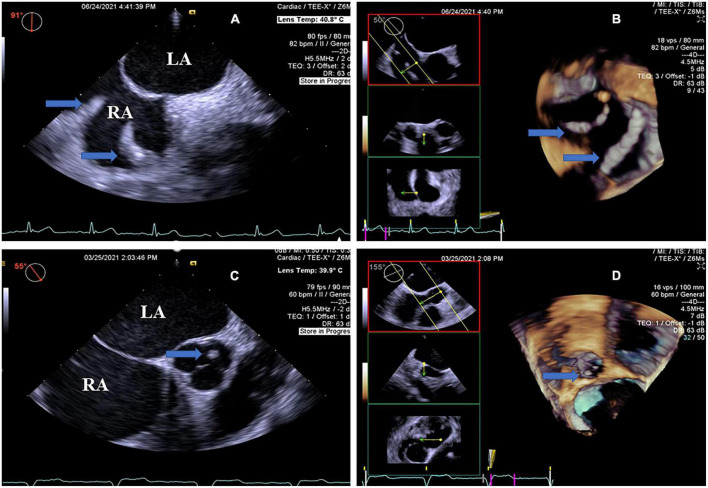
Real-time four-dimensional-TEE evaluating the pacemaker lead-related thrombus and additional thrombus, as compared with 2D-TEE. **(A)** 2D-TEE showing local thickening of the two leads of the pacemaker in the right atrium (arrow) from the double atrial section view and the beaded thrombi (arrow). Note severe spontaneous echo contrast in the left atrium and right atrium. **(B)** RT4D-TEE visualizing the beaded thrombi fused to the pacemaker wire in the right atrium (arrow). **(C)** On the aortic root short axis view, 2D-TEE shows a round mass thrombus in the left aortic sinus attached to the aortic valve (arrow). Note severe spontaneous echo contrast throughout the left atrium and right atrium. **(D)** RT4D-TEE demonstrating an echogenic mass, looks like a round clot thrombus in the left aortic sinus attached to the aortic valve and more clearly than 2D-TEE (arrow) (Movie I–V). Movie I. At a 6-month follow-up, a 64-year-old man revealed a thin layer of thrombus covering the whole surface of the LACbes occluder by RT4D-TEE. Movie II. RT4D-TEE detected a mobile thrombus pedunculated on the face of the Watchman device. Thrombus adhered to the periphery of the occluder. Movie III. RT4D-TEE showed a lead-related floating thrombus in the right atrium. Movie Iv. RT4D-TEE evaluating the beaded thrombi fused to the pacemaker wires in the right atrium. Movie V. RT4D-TEE demonstrating the round mass thrombus in the left aortic sinus from the aortic root short axis view.

In the CIED group, the thrombi attached to the leads of the right chambers were detected by RT4D-TEE in all 17 patients (Figure 3 and Supplementary Video 3). Their thrombi were beaded in three cases (Supplementary Video 4), corded in three cases, and the other 11 cases were clots (Figure 4). In addition, thrombi were found simultaneously in LAA and the left aortic sinus attached to the aortic valve in one case (Supplementary Video 5), whereas four of the lead-attached thrombi detected by RT4D-TEE could not be visualized by 2D-TEE.

(3) Clinical Events and Follow-Up

A total of 40 patients with thrombus were regularly followed up under standardized medical treatment. At the time of finding implant-associated thrombus, the antithrombotic medication regimen consisted of oral anticoagulation in 67.5% (27/40) of patients, OAC plus APT in 5% (2/40) of patients, dual APT in 5% (2/40) of patients, single APT in 2.5% (1/40) of patients, and no antithrombotic therapy in 20% (8/40) of patients. Among those, antithrombotic therapy was mandated in 10% (4/40) of patients due to the need for prolonged triple therapy following percutaneous coronary intervention. Anticoagulants consisting of OAC or single APT were routinely administered to patients following LAAC. 2D-TEE results showed that the change of thrombi after adjusting anticoagulant in 31 patients coincided with that of 4D-TEE. The remaining nine cases of missed diagnosis by 2D-TEE were still unclear.

The incidence of IRT after LAAC was 3.0% (23/760) and clinical outcomes of thrombi were as follows: At 6-month follow-up after adjusting anticoagulant, 4D-TEE results showed that the thrombi were either completely resolved in 30.4% (7/23) of patients and became smaller in 69.6% (16/23) of patients. In the CIED group, the thrombi of three patients attached to the leads illustrated unchanged, which were detected by RT4D-TEE. Of note, their implanted pacemakers had been more than 10 years. The organized thrombi on the pacing lead did not dissolve during our follow-up. The incidence of IRT after CIED was 25% (17/66) and the clinical outcomes of thrombi were as follows: At 6-month follow-up after adjusting anticoagulant, their thrombi were completely resolved in 5.9% (1/17) of cases, became smaller in 64.7% (11/17), and remained unchanged in 29.4% (5/17) of patients as shown by 4D-TEE.

## 4. Discussion

The novel findings of our prospective study were as follows: First, IRT was an overall relatively infrequent event. Second, the detection rate of IRT by RT4D-TEE was 4.8%, significantly higher than that of 3.8% by 2D-TEE. Third, during a 6-month follow-up after adjusting the anticoagulant dosage, RT4D-TEE showed IRT completely dissolved in 20% of patients, became smaller in 67.5% of patients, and showed no change in 12.5% of patients. Compared with 2D-TEE, nine patients still missed the diagnosis. Therefore, RT4D-TEE was superior to 2D-TEE in the accurate diagnosis of IRT.

(1) Implant-related thrombus on occluder detected by RT4D-TEE

In the current study, RT4D-TEE found more scattered IRT on the surface of the occluder and was capable to detect some thrombus masked by mitral regurgitation with more clear imaging than that of 2D-TEE. IRT typically appeared morphologically different, with a large laminar base centering on the atrial facing surface of the device, which limited thrombosis mobility (16–18), and our results were in agreement with the aforementioned reports. The difference in the location where the thrombus stuck between our study and that of Sedaghat et al. (19), following LAAC may be related to a different type of LAA device, device malpositioning, or displacement after the procedure. Bai et al. (17) thought optimal implantation without peri-device gap, individual antithrombotic regimens, and careful monitoring with TEE follow-up could be conducive to the prevention of IRT. In most of the patients, they did not achieve optimal device placement, which was likely to be responsible for thrombus formation (18). In this study, the position of the thrombus stuck to the Watchman device was observed by RT4D-TEE on the central portion, periphery, or edge of the device, respectively. While a thin layer of thrombus was observed covering the surface of the LACbes occluder in two patients. To the best of our knowledge, the Watchman occluder is a plug blocking device, it is implanted slightly deeper into the appendage, leaving a volume of uncovered appendage, which may cause a potential space for the formation of thrombus. Nevertheless, the LACbes occluder is a type of disc blocker device with a slightly larger surface area, which may partly explain the thin layer of thrombus formation. Two patients with AF performed RFCA and LAAC of one stop operation after implanting CIED, and RT4D-TEE detected thrombi on the LAAC device. Nonetheless, OAC or OAC plus APT had been recommended in our present data. In this study, a patient with thrombi on both the occluder and the CIED lead did not appear at the same time.

Another finding from our study was residual shunt after LAAC was detected. Peri-device leak due to incomplete occlusion of the LAA can connect the residual LAA pouch to the systemic circulation (20). The degree of “acceptable” residual peri-device leak (jet of <5 mm in width) was not associated with an increased risk of thromboembolism in a *post hoc* analysis of the Watchman implantation cohort in the PROTECT-AF study (21). In contrast, the presence of a residual shunt of >5 mm typically leads to the continuation of anticoagulation treatment, which may mask the contribution to IRT formation from the residual leak (22, 23). Moreover, the time node of LAA device endothelialization was also an important factor, and it was difficult to form a thrombus after full device endothelialization (21, 24). Pracon et al. (25) demonstrated that IRT was observed early, late, and very late after LAAC in their real-world series, and it was related to patient and procedural characteristics but not to post-implantation DAPT duration. In the LAAC group, at 6-month follow-up after adjusting anticoagulant, 4D-TEE results showed that thrombi were completely resolved in 30.4% (7/23) of cases and the others became smaller. Compared with 2D-TEE, four of them still missed diagnoses. This may be associated with the 2D-TEE can see only a limited angle. Two-dimensional transesophageal echocardiography sections from 0 to 180 degrees do not show the full view of the occluder. Only analyzed by 3D-TEE and 4D-TEE using the software, the entire surface of the occluder can be fully displayed. Thus, it can be seen, thrombus formation on LAA occluder remains a clinical challenge, the evidence regarding the optimal antithrombotic strategy is still under debate as some studies support short-term OAC, whereas others favor single or DAPT only (26, 27).

(2) Implant-related thrombus on CIED lead detected by RT4D-TEE

In our study, RT4D-TEE revealed thrombi throughout the right atrial and ventricular leads and/or right auricula dextra pacing lead by multiple sectional views. The thrombi were non-occlusive, mobile, and either lead attached or in their immediate vicinity among which beaded thrombi were easily missed diagnosis by 2D-TEE. RT4D-TEE demonstrated thrombi on the leads and the other parts of the heart in several patients with an implanted pacemaker for more than 10 years. Even after adjusting the anticoagulant dosage, these thrombi were still difficult to resolve because they had been organized for a long time. Two patients with implanted CIED after RFCA and LAAC of one stop operation. The most frequent indications for CIED were systemic sinus arrest, sick sinus syndrome, or bradycardia. Conversely, Dukkipati et al. (12) demonstrated that after LAAC, most systemic embolism occurred in patients without IRT. At that point, the post-implantation antithrombotic regimen was highly variable with patients receiving OAC. In this situation, the implantation may become a marker rather than the cause, and these patients may be suitable candidates for longer durations of anticoagulation therapy to treat what could be more systemic emboli issues.

The evolving indications and uses for CIEDs have led to a significant increase in the number of implanted devices each year (28). CIED lead-related thrombus may involve only the leads or the whole CIED system, even the extracardiac organs. Although numerous studies have documented an association of systemic embolism (such as cerebral embolism or massive pulmonary embolism) of a patient with a pacemaker (29–31), there are limited data for CIED subjects that our study provides. Generally, TEE is the first convenient tool in visualizing the intra-cardiac portion of the CIED lead. The finding of a free-floating thrombus attached to a pacing lead is much more uncommon. Moreover, it can be more life-threatening due to the high risk of pulmonary embolism. Furthermore, it has been described that the presence of right heart thrombi in acute pulmonary embolism is associated with hemodynamic compromise, right ventricular (RV) hypokinesis, congestive heart failure, poor prognosis, and a higher mortality rate (32). It is evident that massive pulmonary embolism or paradoxical embolism is the cause of the fatal outcome if the thrombus is mobile or free-floating. TEE plays an integral role in evaluating these pacemaker-related complications (33), but the diagnosis of thrombi by 2D-TEE may be technically challenging in patients with small or scattered thrombi. 2D-TEE can only detect parts of the lead from different views, while the whole lead cannot be shown simultaneously in the same section. Ho et al. (4) reported a part of mobile thrombi on transvenous leads was not detected until patients underwent lead extraction, and we think some small clots on the leads are not found by 2D-TEE. Thus, it is crucial to recognize that RT4D-TEE is a novel technique, it can further enhance the detection of lead-related thrombus in one cardiac cycle, and it has the advantage of imaging the lead in multiple views. The detection of thrombi by RT4D-TEE is invaluable by providing direct visualization, measurements of mass, and the ability to assess for associated cardiac involvement. By imaging in more than one RT4D-TEE plane, thrombus can be seen and confirmed, as oscillating intra-cardiac masses on the device lead. In addition, RT4D-TEE has a powerful ability to visualize the entire intra-cardiac route of the leads from the upper vena cava to the RV apex, even the lead in the right auricle, resulting in much higher sensitivity and specificity for this technique. On the other side, the clots on the pacing lead were difficult to resolve in case of longstanding thrombi. In general, anticoagulant drugs were not routinely used after pacemaker implantation, and it took a long time to find out thrombus or detect it by chance during an examination, as we have studied. But in that analysis, the number of patients included was relatively small, the thrombus attached to the lead was found at the time of TEE examination, limited imaging was available, and most importantly.

Notably, the volume of RA in the CIED group was much larger than in the LAAC group. Herein, we hypothesize that the risk of thrombus formation may be increased because the volume of RA has progressed to blood stasis in these patients. In reviewing the development of significant tricuspid regurgitation (TR) following the CIED placement, a number of different mechanisms of RV intra-cardiac lead-related TR should be considered, which might explain the RA dilation and AF occurrence. The aforementioned predisposing factor, combined with chambers dilated and evidence of hemodynamic impairment with signs of congestive heart failure, might have contributed to the CIED lead thrombus.

## 5. Limitations

First, this was not a randomized study, the sample size was relatively small, and the follow-up was not long enough to predict long-term improvement. Second, it could not further divide the patients into subgroups by different types of LAAC occluders and/or electronic devices. Third, we did not compare RT4D-TEE for the detection of IRT with other modalities such as cardiac computed tomography angiography and nuclear studies. However, these limitations may not affect echocardiographic findings. A multicenter study should be scheduled in the future to address these issues.

## 6. Conclusion

In summary, the accurate detection rate of IRT by RT4D-TEE was 4.8%, higher than that of 3.8% by 2D-TEE. The limitations of 2D-TEE include that cannot show the full view of the occluder and that reveals the whole lead simultaneously in one section. Thus, RT4D-TEE is a sensitive method. Imaging and some clinical features are also quite different between IRT on occluder and CIED lead.

## Data availability statement

The original contributions presented in this study are included in the article/Supplementary material, further inquiries can be directed to the corresponding authors.

## Ethics statement

The studies involving human participants were reviewed and approved by the Ethics Committee of Xinhua Hospital Affiliated to Shanghai Jiao Tong University School of Medicine. The patients/participants provided their written informed consent to participate in this study.

## Author contributions

YY participated in conception and design. Y-GL and Y-PW provided the administrative support. Y-HC and RZ provided the materials or patients. YS, TW, and ZW performed the collection and assembly of data. X-LT and C-QG performed the data analysis and interpretation. All authors performed the article writing and final approval of article.

## References

[1] Wazni O Saliba W Hussein A. Device-related thrombus after left atrial appendage occlusion. *JACC.* (2021) 78:314–6. 10.1016/j.jacc.2021.05.028 34294268

[2] Saw J Tzikas A Shakir S Gafoor S Omran H Nielsen-Kudsk J Incidence and clinical impact of device-associate thrombus and peri-device leak following left atrial appendage closure with the amplatzer cardiac plug. *JACC Cardiovasc Interv.* (2017) 10:391–9. 10.1016/j.jcin.2016.11.029 28231907

[3] Alkhouli M Busu T Shah K Osman M Alqahtani F Raybuck B. Incidence and clinical impact of device-related thrombus following percutaneous left atrial appendage occlusion: a meta-analysis. *JACC Clin Electrophysiol.* (2018) 4:1629–37. 10.1016/j.jacep.2018.09.007 30573129

[4] Ho G Bhatia P Mehta I Maus T Khoche S Pollema T Prevalence and short-term clinical outcome of mobile thrombi detected on transvenous leads in patients undergoing lead extraction. *JACC Clin Electrophysiol.* (2019) 5:657–64. 10.1016/j.jacep.2019.01.007 31221351

[5] Korkeila P Saraste M Nyman K Koistinen J Lund J Airaksinen K. Transesophageal echocardiography in the diagnosis of thrombosis associated with permanent transvenous pacemaker electrodes. *Pacing Clin Electrophysiol.* (2006) 29:1245–50. 10.1111/j.1540-8159.2006.00519.x 17100678

[6] Cochet H Iriart X Sridi S Camaioni C Corneloup O Montaudon M Left atrial appendage patency and device-related thrombus after percutaneous left atrial appendage occlusion: a computed tomography study. *Eur Heart J Cardiovasc Imag.* (2018) 19:1351–61. 10.1093/ehjci/jey010 29415203

[7] Hindricks G Potpara T Dagres N Arbelo E Bax J Blomström-Lundqvist C 2020 ESC guidelines for the diagnosis and management of atrial fibrillation developed in collaboration with the European Association for Cardio-Thoracic Surgery (EACTS): the task force for the diagnosis and management of atrial fibrillation of the European Society of Cardiology (ESC) developed with the special contribution of the European Heart Rhythm Association (EHRA) of the ESC. *Eur Heart J.* (2021) 42:373–98. 10.1093/eurheartj/ehab648 32860505

[8] Munawar D Chan J Emami M Kadhim K Khokhar K O’Shea C Magnetic resonance imaging in non-conditional pacemakers and implantable cardioverter-defibrillators: a systematic review and meta-analysis. *Europace.* (2020) 22:288–98. 10.1093/europace/euz343 31995177

[9] Meier B Blaauw Y Khattab A Lewalter T Sievert H Tondo C EHRA/EAPCI expert consensus statement on catheter-based left atrial appendage occlusion. *Euro Interv.* (2015) 10:1109–25. 10.4244/EIJY14M09_18 25169595

[10] Korsholm K Jensen J Nørgaard B Nielsen-Kudsk J. Detection of device-related thrombosis following left atrial appendage occlusion: a comparison between cardiac computed tomography and transesophageal echocardiography. *Circ Cardiovasc Interv.* (2019) 12:e008112. 10.1161/CIRCINTERVENTIONS.119.008112 31514523

[11] Di Vincenzo A Rizzo A Russo L Mioni R. Pacemaker-associated thrombosis in ongoing therapy with edoxaban tosylate. *J Thromb Thrombolysis.* (2018) 46:549–50. 10.1007/s11239-018-1733-z 30182222

[12] Dukkipati S Kar S Holmes D Doshi S Swarup V Gibson D Device-related thrombus after left atrial appendage closure. *Circulation.* (2018) 138:874–85. 10.1161/CIRCULATIONAHA.118.035090 29752398

[13] Kar S Doshi S Sadhu A Horton R Osorio J Ellis C Primary outcome evaluation of a next-generation left atrial appendage closure device: results from the PINNACLE FLX trial. *Circulation.* (2021) 143:1754–62. 10.1161/CIRCULATIONAHA.120.050117 33820423

[14] Main M Fan D Reddy V Holmes D Gordon N Coggins T Assessment of device-related thrombus and associated clinical outcomes with the WATCHMAN left atrial appendage closure device for embolic protection in patients with atrial fibrillation (from the PROTECT-AF trial). *Am J Cardiol.* (2016) 117:1127–34. 10.1016/j.amjcard.2016.01.039 26993976

[15] Korsholm K Nielsen K Jensen J Jensen H Andersen G Nielsen-Kudsk J. Transcatheter left atrial appendage occlusion in patients with atrial fibrillation and a high bleeding risk using aspirin alone for postimplant antithrombotic therapy. *EuroIntervention.* (2017) 12:2075–82. 10.4244/EIJ-D-16-00726 27973336

[16] Saw J Nielsen-Kudsk J Bergmann M Daniels M Tzikas A Reisman M Antithrombotic therapy and device-related thrombosis following endovascular left atrial appendage closure. *JACC Cardiovasc Interv.* (2019) 12:1067–76. 10.1016/j.jcin.2018.11.001 31103535

[17] Bai Y Xue X Duenninger E Muenzel M Jiang L Keil T Real-world survival data of device-related thrombus following left atrial appendage closure: 4-year experience from a single center. *Heart Vessel.* (2019) 34:1360–9. 10.1007/s00380-019-01364-7 30820642

[18] Aminian A Schmidt B Mazzone P Berti S Fischer S Montorfano M Incidence, characterization and clinical impact of device-related thrombus following left atrial appendage occlusion in the prospective global AMPLATZER Amulet observational study. *JACC Cardiovasc Interv.* (2019) 12:1003–14. 10.1016/j.jcin.2019.02.003 31103540

[19] Sedaghat A Schrickel J Andrie R Schueler R Nickenig G Hammerstingl C. Thrombus formation after left atrial appendage occlusion with the Amplatzer Amulet device. *JACC Clin Electrophysiol.* (2017) 3:71–5. 10.1016/j.jacep.2016.05.006 29759698

[20] Kanderian A Gillinov A Pettersson G Blackstone E Klein A. Success of surgical left atrial appendage closure: assessment by trans esophageal echocardiography. *J Am Coll Cardiol.* (2008) 52:924–9. 10.1016/j.jacc.2008.03.067 18772063

[21] Sivasambu B Arbab-Zadeh A Hays A Calkins H Berger R. Delayed endothelialization of watchman device identified with cardiac CT. *J Cardiovasc Electrophysiol.* (2019) 30:1319–24. 10.1111/jce.14053 31257658

[22] Staubach S Schlatterbeck L Mörtl M Strohm H Hoppmann P Laugwitz K Long-term transesophageal echocardiography follow-up after percutaneous left atrial appendage closure. *Heart Rhythm.* (2020) 17:728–33. 10.1016/j.hrthm.2019.12.004 31841716

[23] Nguyen A Gallet R Riant E Deux J Boukantar M Mouillet G Peridevice leak after left atrial appendage closure: incidence, risk factors, and clinical impact. *Can J Cardiol.* (2019) 35:405–12. 10.1016/j.cjca.2018.12.022 30935631

[24] Simrad T Jung R Lehenbauer K Piayda K Pracoń R Jackson G Predictors of device-related thrombus following percutaneous left atrial appendage occlusion. *J Am Coll Cardiol.* (2021) 78:297–313. 34294267 10.1016/j.jacc.2021.04.098

[25] Cruz-González I Korsholm K Trejo-Velasco B Thambo J Mazzone P Rioufol G Procedural and short-term results with the new Watchman FLX left atrial appendage occlusion device. *JACC Cardiovasc Interv.* (2020) 13:2732–41. 10.1016/j.jcin.2020.06.056 33189641

[26] Patti G Sticchi A Verolino G Pasceri V Vizzi V Elvis B Safety and efficacy of single versus dual antiplatelet therapy after left atrial appendage occlusion. *Am J Cardiol.* (2020) 134:83–90. 10.1016/j.amjcard.2020.08.013 32892987

[27] Asmarats L O’Hara G Champagne J Paradis J Bernier M O’Connor K Short-term oral anticoagulation versus antiplatelet therapy following transcatheter left atrial appendage closure. *Circ Cardiovasc Interv.* (2020) 13:e009039. 10.1161/CIRCINTERVENTIONS.120.009039 32772571

[28] Karavidas A Lazaros G Matsakas E Kouvousis N Samara C Christoforatou E Early pacemaker lead thrombosis leading to massive pulmonary embolism. *Echocardiography.* (2004) 21:429–32. 10.1111/j.0742-2822.2004.03078.x 15209722

[29] Böhm A Bányai F Komáromy K Pintér A Préda I. Cerebral embolism due to a retained pacemaker lead: a case report. *PACE.* (1998) 21:629–30. 10.1111/j.1540-8159.1998.tb00111.x 9558700

[30] Arslan S Gundogdu F Bozkurt E. Permanent pacemaker lead thrombosis leading to recurrent pulmonary embolism. *Heart.* (2006) 92:597. 10.1136/hrt.2005.075192 16614270 PMC1860936

[31] Buttigieg J Asciak R Mallia Azzopardi C. Pacemaker lead-associated thrombosis in cardiac resynchronisation therapy. *BMJ Case Rep.* (2015) 7:bcr2015210314. 10.1136/bcr-2015-210314 26153289 PMC4499717

[32] Torbicki A Galié N Covezzoli A Rossi E Rosa M Goldhaber S. ICOPER study group. right heart thrombi in pulmonary embolism: results from the International cooperative pulmonary embolism registry. *J Am Coll Cardiol.* (2003) 41:2245–51. 10.1016/S0735-1097(03)00479-0 12821255

[33] Almomani A Siddiqui K Ahmad M. Echocardiography in patients with complications related to pacemakers and cardiac defibrillators. *Echocardiography.* (2014) 31:388–99. 10.1111/echo.12483 24341293

